# Bullying, Depression, and Suicidal Ideation Among Adolescents in the Fujian Province of China: A Cross-sectional Study

**DOI:** 10.1097/MD.0000000000002530

**Published:** 2016-02-08

**Authors:** Lingyao Hong, Lan Guo, Hong Wu, Pengsheng Li, Yan Xu, Xue Gao, Jianxiong Deng, Guoliang Huang, Jinghui Huang, Ciyong Lu

**Affiliations:** From the Department of Medical statistics and Epidemiology, School of Public Health, Sun Yat-sen University (LH, LG, HW, PL, CL); Center for ADR monitoring of Guangdong (YX, XG, JD, GH, JH); and Epidemiology Research Unit, Clinical trials Center, The First Affiliated Hospital, Sun Yat-sen University, Guangzhou, China (LH).

## Abstract

The relationship of bullying with suicidal ideation among adolescents is controversial. Although depression has been related to bullying and suicidal ideation, little is known about the combined impacts of depression and bullying on suicidal ideation.

A sample of 20,509 high school students from Fujian Province were selected randomly by multistage stratified sampling. All participants completed an adolescent health status questionnaire. Three categories of bullying were assessed: perpetration, victimization, and both (victimization and perpetration). The associations of these 3 categories of bullying with depression and their interaction with suicidal ideation were examined in logistic models.

After adjustment for potential confounders, all 3 categories of bullying (perpetration, victimization, and both) were related to increased risk of suicidal ideation with odds ratios (ORs) of 1.66 (95% confidence interval [CI] 1.20–2.30), 2.74 (95% CI 2.29–3.29), and 2.83 (95% CI 2.27–3.52), respectively. There was an interaction between depression and bullying (*P* = 0.001). Subgroup analyses showed a stronger association between perpetration and suicidal ideation in students with depression (odds ratio [OR] 2.97; 95% CI 1.44–6.09) than in those without depression (OR 1.65; 95% CI 1.19–2.28). The association between victimization and suicidal ideation was weaker in students with depression (OR 1.49; 95% CI 1.07–2.07) than in those without (OR 2.69; 95% CI 2.24–3.23). The association of both victimization and perpetration with suicidal ideation was weaker in students with depression (OR 2.22, 95% CI 1.43–3.47) than those without (OR 2.78; 95% CI 2.23–3.47).

We observed an independent association of bullying with increased risk of suicidal ideation among adolescent students, and this association was affected by depression. Prospective studies should be conducted to confirm these findings.

## INTRODUCTION

Suicide is a greatly important public health concern worldwide. Adolescent students experience great pressure from social developmental transitions and are at high risk of suicide.^1^ Suicidal ideation has been reported to be an important suicide risk factor in adolescence.^2^ Currently, suicidal ideation is common in adolescence in both developing and developed countries.^3–5^ The causes of suicidal ideation are multifactorial, including factors related to biological, psychological, cognitive, school, family, and social risk domains.^6–9^

Bullying among school-aged youth is a common issue worldwide. Recent attention has focused on the association between bullying and suicidal ideation in adolescent students. Three categories of bullying include perpetration, victimization, and both (victimization and perpetration). Most previous studies have found that all 3 types of bullying were related to increased risk of suicidal ideation with the strongest risks in victim perpetrators.^10–12^ However, these significant associations have not been confirmed by other studies,^13,14^ and Liang et al reported that perpetrators and victims, but not victim perpetrators, were more likely to think about committing suicide.^15^ The reason for these discrepant study results is not clear, but may be due to potential confounding factors, relatively small or selective samples, and the methodology of measuring bullying.

Several studies have shown that suicidal ideation is associated with depression in adolescents,^16,17^ and adolescents involved in bullying are more likely to experience depression.^18,19^ However, it is unknown whether the association of bullying with suicidal ideation is different in adolescents with and without depression. In this cross-sectional study, we evaluated the association of bullying with suicidal ideation among adolescent students and investigated whether this association varied in relation to the presence of depression.

## METHODS

### Participants

This was a cross-sectional study in adolescent students from the Fujian Province in southeastern China. Subjects were selected by a 3-stage, stratified sampling method. First, we categorized Fujian Province cities within 1 of 3 economic status levels, and then 2 cities in each category were selected randomly, yielding a total of 6 included cities. Second, we categorized the high schools in Fujian Province as vocational high schools, senior high schools, or junior high schools. Based on the proportions of these 3 types of schools, we selected 2 vocational high schools (ie, grade 7–12), 4 senior high schools (ie, grade 7–9), and 6 junior high schools (ie, grade 10–12) randomly in each of the 6 cities, enrolling a total of 72 schools. Finally, we used a simple random sampling method to choose 2 classes from each grade at each school. All 20,900 subjects studying in those classes were invited to complete an adolescent health status questionnaire.

### Data Collection

During class time, students were asked to fill out self-administered questionnaires anonymously in the absence of school teachers to avoid information bias. In each classroom, we arranged for 2 investigators who had received intensive training on how to administer the questionnaire to supervise the survey. We collected all data in 2011 and 2012.

### Ethical Statement

Students were informed of the study objectives and procedures. Participation was voluntary, and questionnaires were anonymous to protect student privacy. We obtained written consent from 1 parent per student and from all students. The Institutional Review Board of the School of Public Health, Sun Yat-sen University (Guangzhou, China) approved the study.

### Measures

#### Suicidal Ideation

Suicidal ideation was defined as thinking about committing suicide during the last 12 months, based on the response to the question: “Did you think about committing suicide during the past 12 months?” Coding: (0 = no, 1 = yes).

#### Bullying Behaviors

Bullying perpetration was assessed with 6 questions. Students were asked how frequently they had bullied others in the past month. The following bullying questions were posed: “Have you ever beaten, kicked, pushed someone, or kept someone in the house?”; “Have you ever teased someone maliciously?”; “Have you ever isolated someone or excluded someone from group activities intentionally?”; “Have you ever played sexual jokes on someone or made sexual gestures in front of someone?”; “Have you ever blackmailed someone for money?”; and “Have you ever bullied someone in other ways?” Each question was coded: 1 = never, 2 = sometimes (1 or 2 times), 3 = often (more than 3 times). Participants reporting an answer of “often” to at least 1 of the questions were categorized as perpetrators.^20^

Bullying victimization was assessed with 6 parallel questions. Students were asked how frequently they had been bullied in the past month. The being bullied items were the following: “Have you ever been beaten, kicked, pushed, or kept in the house?”; “Have you ever been teased maliciously?”; “Have you ever been isolated or excluded from group activities intentionally?”; “Have you ever been made fun of with sexual gestures or jokes?”; “Have you ever been blackmailed for money?”; and “Have you ever been bullied in other ways?” Each question was coded: 1 = never, 2 = sometimes (1 or 2 times), and 3 = often (more than 3 times). Participants reporting an answer of “often” to at least 1 of the questions were categorized as victims.^21^ Thus, there were 4 types of subjects: perpetration only, victimization only, both (victimization and perpetration), and neutrals who were not involved in frequent bullying.

#### Depression

We used the Center for Epidemiology Scale for Depression (CES-D) to measure whether participants had depression. The CES-D was developed for use in studies on the epidemiology of depressive symptomatology and it has been shown to have good reliability and validity in adolescent and young adult populations.^22,23^ The Chinese version of the CES-D has been validated and used extensively.^24–27^ The CES-D includes 20 questions, on which participants rate the frequency of depressive symptoms over the past week. Each question is coded: 0 = rarely or never, 1 = 1 to 2 days per week, 2 = 3 to 4 days per week, 3 = 5 to 7 days per week. Four of the items (items 4, 8, 12, and 16) are reverse coded, and the total score ranged from 0 to 60. We adopted a cut off value of >28 (95th percentile) out of 60 to define depression. Data from questionnaires with fewer than 17 items completed were excluded.

#### Demographic Variables

We collected demographic information, including sex, grade, age, academic pressure, living arrangement, family economic status, relationships with teachers, classmates and families, and smoking. Students rated their academic pressure and family economic status from low to high and relationships from poor to good. Smoking was defined as ever having smoked during one's lifetime, based on the response to the question: “Have you ever smoked during your life time?” Coding: (0 = no, 1 = yes).

### Statistical Analysis

We used SPSS version 16.0 (SPSS Inc, Chicago, IL) to analyze all data. Data were described as number (%) or mean ± SD. We used univariate and multivariate logistic models to evaluate associations of demographic characteristics with suicidal ideation. Unadjusted and adjusted logistic regression models were used to calculate odds ratios (ORs) and 95% confidence intervals (CIs) for associations among bullying and depression with suicidal ideation. To investigate whether the association between bullying and suicidal ideation differs between students with and without depression, we tested the interaction factors of bullying and depression. Variables with *P*-value <0.05 in the multivariate model (Table 1) were selected as potential confounders for the adjusted model. Finally, we performed subgroup analyses to measure the relationship of bullying with suicidal ideation in students with or without depression. We excluded missing data when doing multivariable analysis. *P*-value <0.05 was considered significant.

**TABLE 1:**
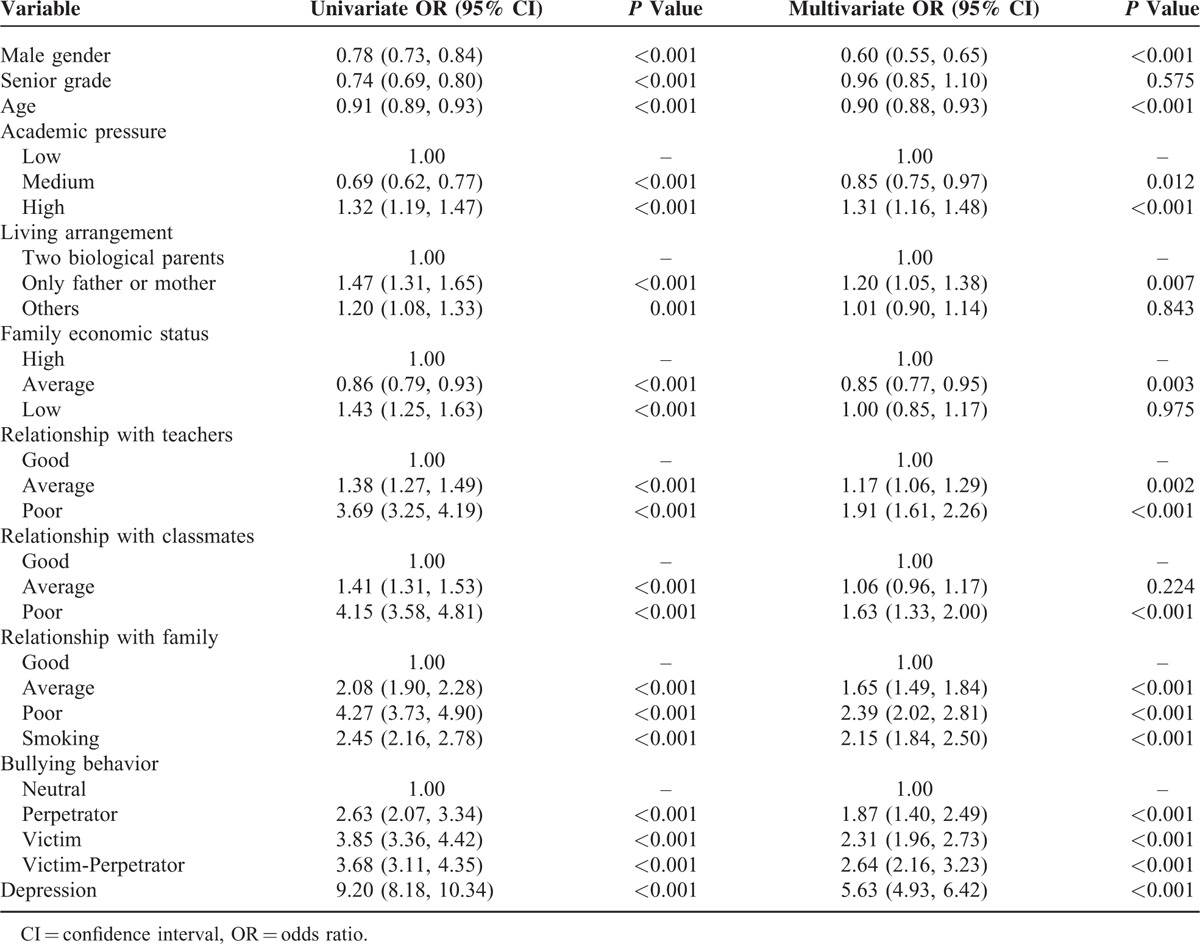
Associations Between Demographic Characteristics and Suicidal Ideation by Univariate and Multivariate Logistic Regression Analysis

## RESULTS

### Demographic Characteristics

Among the 20,900 students who participated, 20,511 valid questionnaires (98.1%) were recovered and analyzed. The participants’ basic demographic characteristics are summarized in Table 2. The mean age was 16.3 ± 2.0 years, 54.6% were females, and 50.8% were in their junior year of school. Regarding academic pressure, 40.4% gave high ratings and 16.3% gave low ratings. The majority (77.6%) lived with 2 biological parents, and 8.8% rated their family economic status as low. Poor relationships were reported with teachers, classmates, and families by 6.7%, 4.0%, and 4.6% of students, respectively, and 5.9% were smokers. The proportions of perpetrators, victims, and victim perpetrators were 1.5%, 4.5%, and 3.0%, respectively. Finally, 17.2% reported suicidal ideation during the last 12 months, and 6.5% had depression according to the CES-D.

**TABLE 2:**
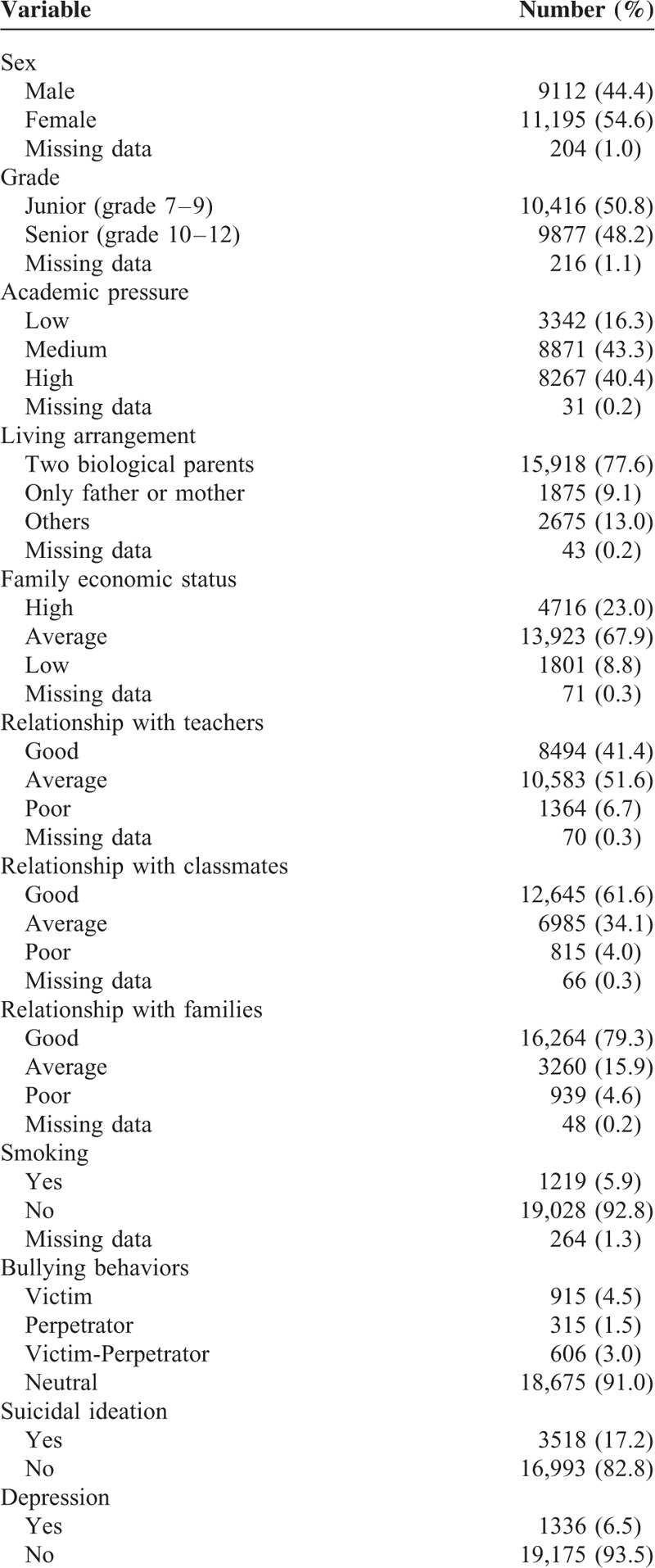
Demographic Characteristics (N = 20,511)

### Associations Between Demographic Characteristics and Suicidal Ideation

Our univariate and multivariate logistic models for suicidal ideation are presented in Table 2. In the univariate models, suicidal ideation correlated with sex, grade, age, academic pressure, living arrangement, family economic status, relationships with teachers, classmates, and families, smoking, bullying, and depression. All of the significant factors in the univariate models except for grade related significantly to suicidal ideation in the multivariate model.

### Association of Bullying, Depression, and Their Interactions With Suicidal Ideation

As shown in Table 3, all 3 forms of being involved with bullying (perpetration, victimization, and both) were related to increased risk of suicidal ideation in both unadjusted and adjusted models. After adjustment for potential confounders, the ORs of perpetrators, victims, and victim perpetrators having suicidal ideation were 1.66 (95% confidence interval [CI] 1.20–2.30), 2.74 (95% CI 2.29–3.29), and 2.83 (95% CI 2.27–3.52), respectively. Students with depression had a higher probability of suicidal ideation with an odds ratio (OR) of 6.27 (95% CI 5.39–7.29). Furthermore, a significant interaction was identified between bullying and depression in both unadjusted and adjusted models (*P*-interaction = 0.001 in both models).

**TABLE 3:**
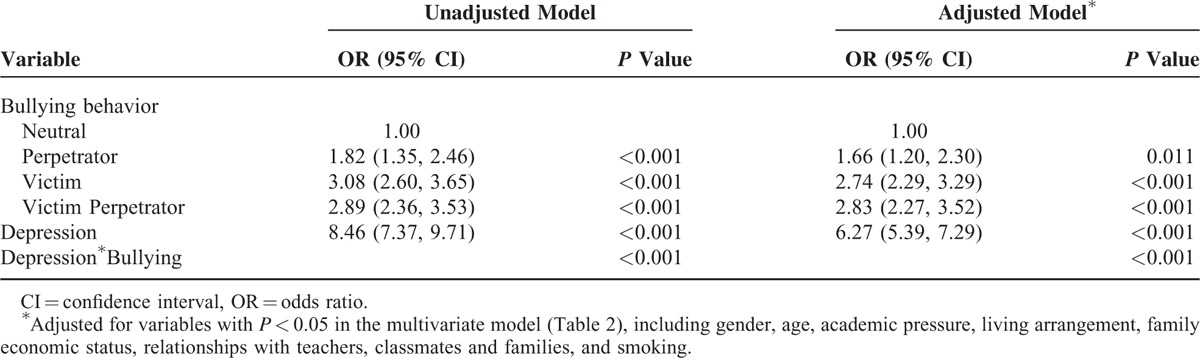
Association of Bullying, Depression, and Their Interaction With Suicidal Ideation

### Association of Bullying and Suicidal Ideation Stratified by Depression

In Table 4, we did subgroup analysis stratified by depression. The association between perpetration and suicidal ideation was stronger in students with depression (OR 2.97; 95% CI 1.44–6.09) than in those without depression (OR 1.65; 95% CI 1.19–2.28). The association between victimization and suicidal ideation was weaker in students with depression (OR 1.49; 95% CI 1.07–2.07) than in those without (OR 2.69; 95% CI 2.24–3.23). The association of both victimization and perpetration with suicidal ideation was weaker in students with depression (OR 2.22; 95% CI 1.43–3.47) than those without (OR 2.78; 95% CI 2.23–3.47).

**TABLE 4:**
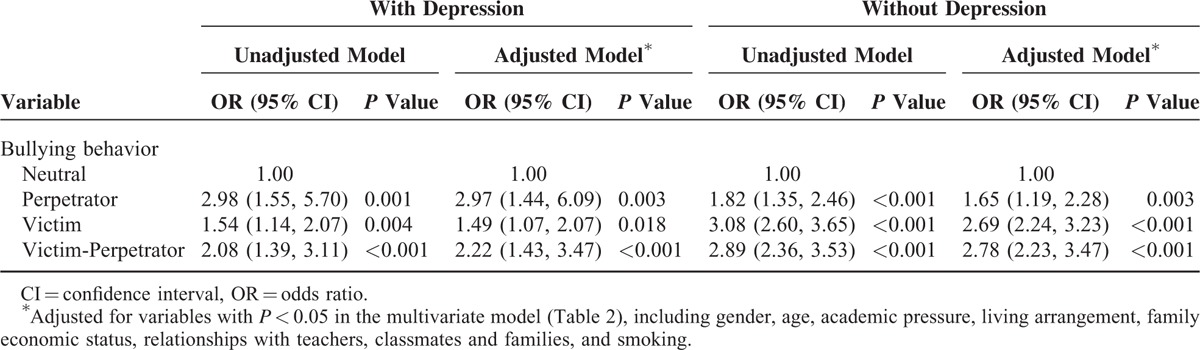
Association of Bullying With Suicidal Ideation Stratified by Depression

## DISCUSSION

We found that all 3 forms of being involved in bullying (perpetration, victimization, and both) independently associated with increased risk of suicidal ideation. Furthermore, there was a significant interaction between depression and bullying, suggesting that depression may modify the influence of bullying on suicidal ideation.

Previous studies have focused on the association between different types of bullying and suicidal ideation in adolescence. A study by Hepburn et al showed that students involved in any type of bullying were more likely to report suicidal ideation. After adjusting for sex, race, and grade, they found that victim perpetrators were at the highest risk among the 3 types of bullying with ORs for suicidal ideation of 3.78 (95% CI: 2.86–4.99).^28^ Similar results were reported by Brunstein et al.^19^ In our study, the results were similar to those of Hepburn et al and Brunstein et al after adjusting for confounding factors. However, some other studies have reported contrary findings. Park et al reported no association between bullying and suicidal ideation,^14^ and Liang et al reported that perpetrators and victims, but not victimperpetrators, were more likely to think about committing suicide.^15^ The differences between our results and those of other studies might be due to limited sample size and the methodology of measuring bullying. Park et al surveyed only 1312 students in their study, and Liang et al used 2 simple unstructured questions to measure bullying status of participants. Recently, a meta-analysis of 47 studies showed that involvement in all 3 types of bullying was associated with suicidal ideation, and the risk in victim perpetrators was highest.^29^ It is difficult to apply the findings of this meta-analysis to our study, since only 3 of the studies included were conducted in China, and those only examined the association between victimization and suicidal ideation. Furthermore, most of the 47 studies in the meta-analysis did not control for some well-established risk factors when calculating ORs, such as age, sex, family economic status, or depression. Our study is based on a large adolescent sample from China, showed that high school students involved in bullying are more likely to have suicidal ideation, and the most troubled adolescents are victim perpetrators.

To the best of our knowledge, this study is the first to investigate the interaction between depression and bullying with suicidal ideation. Among students with depression, we found that the association between perpetration and suicidal ideation was stronger in students with depression than in those without depression. As depression *per se* was a significant independent risk factor for suicidal ideation,^16,17^ our results indicated an additive synergism between depression and perpetration on suicidal ideation. However, the associations of victimization and both (victimization and perpetration) with suicidal ideation were weaker in students with depression, compared with those without. This unexpected finding might be due to selection bias because our study was school based. Qurioga et al reported that depression increased the risk of students dropping out of school by 2.75-fold.^30^ Thus, we may have under-sampled depressed students in this study. In addition, depressed students who are bullied at school may be more likely to withdraw from school than perpetrators and neutrals who are not involved regularly in bullying. If so, the ORs of bullying with suicidal ideation among depressed students in our study may have underestimated the associations, especially among victims and victim perpetrators.

Although these results enhance our understanding of the etiology of suicidal ideation among adolescents, this study has some limitations. First, its cross-sectional design limits analysis to statistical relationships, and prospective studies will be required to examine cause-effect relationships. Second, our study was a school-based study, which may lead to selection bias, and community-based study will be required. Third, measurements of bullying were self-reported. Although bullying was defined with detailed questions, and this method is widely accepted, the answers could still be subjectively biased.

In conclusion, bullying (perpetration, victimization, and both) increases the probability of suicidal ideation among Chinese adolescent students, and the risk was highest in victim perpetrators. Depression modified this relationship. Subgroup analyses showed that, in students with depression, the association of perpetration with suicidal ideation was stronger, and the association of victimization and both (victimization and perpetration) with suicidal ideation was weaker compared with those without depression.

Suicide has become the third leading cause of death in adolescence, and more attention should be focused on risk factors and prevention of suicidal ideation.^31^ Even though we do not know the precise mechanisms for the relationships found in this study, the results are important for schools, families, and social agencies who strive to support adolescents involved in bullying.
